# Viral diseases of the eye

**Published:** 2020-03-30

**Authors:** Jeremy Hoffman, Allen Foster

**Affiliations:** 1 Clinical Research Fellow: International Centre for Eye Health, London School of Hygiene & Tropical Medicine, UK.; 2 Professor, International Centre for Eye Health, London School of Hygiene & Tropical Medicine, UK.

**Viruses are tiny particles that cannot replicate – or survive for very long – outside the cells of their host organism; yet they remain an ongoing risk to our health and our eye health.**

**Figure:**
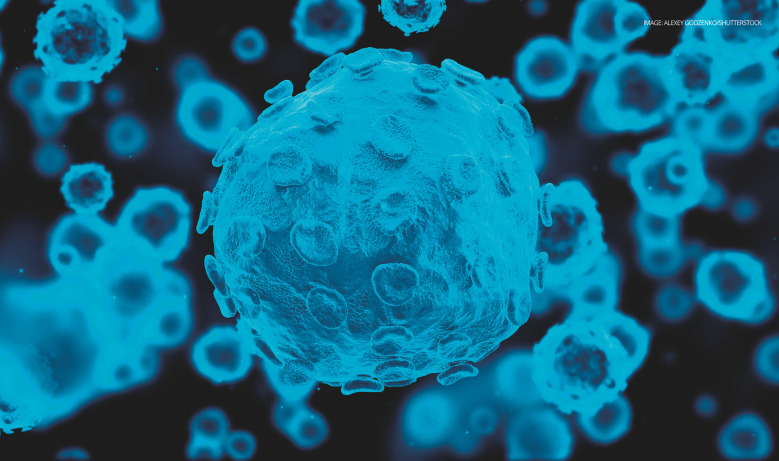
Herpes simplex virus.

Globally, viral infections of the eye are common. They are a significant cause of acute red eye and visual loss. Any part of the eye and adnexa – from the eyelids to the retina and optic nerve – can be affected by viral disease. Some ocular viral infections, such as viral conjunctivitis due to adenovirus or influenza virus, are short lived, with limited ocular complications. However, other viral infections can cause serious complications, such as corneal scarring from stromal keratitis due to herpes simplex virus (HSV) or retinal detachment resulting from cytomegalovirus (CMV) retinitis.

Certain viral eye diseases demonstrate clear clinical signs that enable diagnosis (such as the dendritic ulcers due to HSV or the unilateral trigeminal nerve distribution of herpes zoster ophthalmicus). In other cases (for example follicular conjunctivitis or uveitis), a variety of different viral infections may be responsible for the same clinical signs. Making a specific diagnosis can therefore be very challenging.

Antibiotics are not effective against viruses, but evidence-based anti-viral treatments exist for several viral infections, including herpes simplex (HSV), varicella zoster (VZV), cytomegalovirus (CMV) and human immune-deficiency virus (HIV).

**Figure 1:**
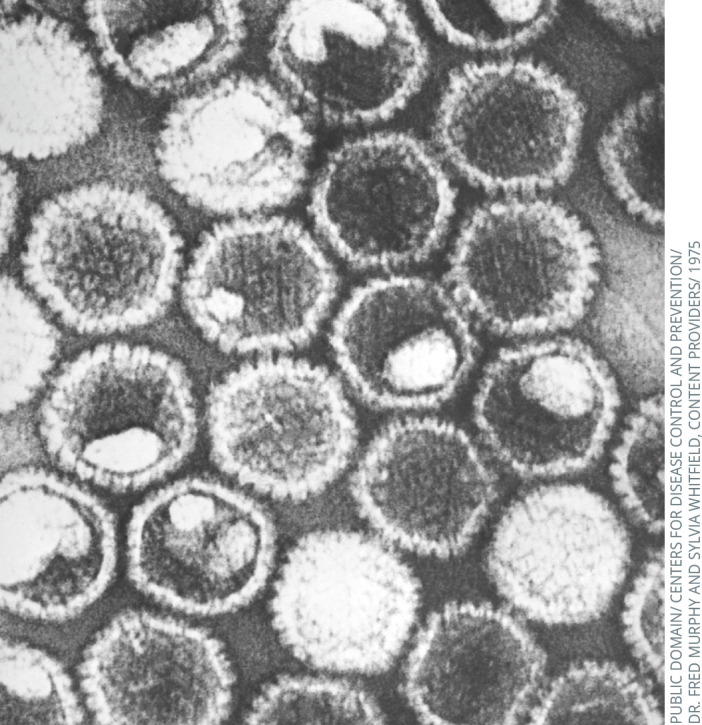
Electron micrograph image of several herpes simplex viruses, showing the lipid envelopes (studded with glycoprotein ‘spikes’). Inside each envelope is the nucleocapsid, which houses the viral DNA.

## What you will learn

In this issue, we present reviews of eye disease due to herpes simplex and herpes zoster. These are both DNA viruses (see p. 67) and tend to lie dormant in nerve ganglia. Adenovirus is another DNA virus; it usually causes an infectious follicular conjunctivitis. There are also review articles on viral diseases which have come into more prominence in the last two to three decades: HIV, CMV and, more recently, Ebola and Zika. Finally, we have included an article that reviews our current understanding of the available treatments for ocular viral infections.

The aim of this issue is to present an overview of the common and important ocular viral infections, how a clinical diagnosis can be made, and the steps that can be taken to prevent and treat ocular viral infections in order to reduce eye disease and visual loss. If you find it useful, please share it with your colleagues and team members.

**How viruses work**

**Figure 2:**
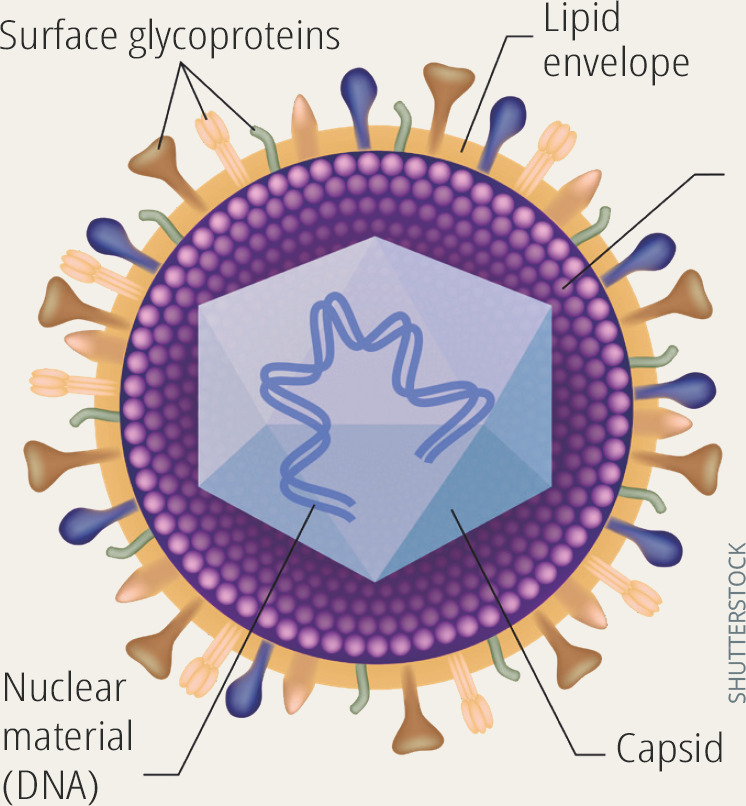
Simplified diagram of the herpes simplex virus.

Viruses are very small infectious agents (Figure 2). They consist of:

- Genetic material (RNA or DNA)
- A protein coat (the capsid) that surrounds or protects the genetic material
- A lipid envelope around the capsid, studded with unique surface proteins (glycoproteins). Some viruses do not have an envelope.

Viruses are very simple organisms and can only multiply within the living cells of a host organism, such as a human being. A virus will enter the cell and trick it into making copies of the virus until the cell bursts and releases the copies, often destroying the cell in the process.

The viruses – known as viral particles or virions when outside a cell – can then invade other cells or be transmitted to another host. The main mechanisms of transmission are:

- Exchange of bodily fluids (e.g., via blood transfusion, kissing or sexual intercourse)
- Inhalation of airborne viral particles (after an infected person coughs or sneezes)
- Transfer of viral particles from contaminated surfaces to the eyes, nose or mouth, usually via the hands. Surfaces – including ophthalmic equipment – can become contaminated when an infected person coughs or sneezes, or touches the surface after touching their nose, mouth or eyes
- Contaminated surgical instruments that come into contact with body tissues and fluids during surgery.

Viruses cause disease in two ways:

- The direct action of the virus on cells. For example, herpes simplex dendritic ulcer.
- The body's immune response to the virus and/or virus-infected cells. For example, herpes simplex disciform endotheliitis (p. 69).

**The immune response**

The body's immune system responds to a viral infection by making specific antibodies (the immunoglobulins IgM and IgG) designed to bind either to the unique viral surface proteins, or to viral fragments that remain outside the host cell. The antibody can help to mark the virus or infected cell for destruction by the body's T lymphocyte cells (also known as killer T cells). Once the viral infection has been halted, the memory to make antibodies remains in the immune system of the host, which allows the immune system to recognise the same virus immediately and act to stop a new infection.

Immunisation takes advantage of this fact. It involves exposing the host to a non-pathogenic version of the virus: so that the person doesn't become ill, but the immune system produces antibodies against future exposure to a pathogenic version of the virus. This approach is used successfully with some viruses, such as measles and rubella, which are considered ‘stable’. However, with other viruses, such as HIV, the surface proteins are continually changing, so that antibodies do not work effectively. The immune system struggles to fight such virus infections and immunisation is therefore problematic.

**Prevention**

Some viral infections can also be prevented by reducing transmission between people. Avoiding unprotected sexual intercourse reduces the risk of transmission of human immunodeficiency virus (HIV) and herpes simplex virus 1 (HSV-1). Regular and effective handwashing helps to prevent the transmission of some viruses e.g., adenovirus.

In a hospital or clinic setting, you can reduce transmission between doctor and patient, and from patient to patient, by practising good hygiene. This includes:

- Close attention to handwashing (scrubbing)
- Cleaning of all surfaces, equipment and instruments using appropriate methods and antiviral solutions
- Wearing protective clothing such as gloves and, in some circumstances, a mask.

*Editorial team*

